# The Induction of IgM and IgG Antibodies against HLA or MICA after Lung Transplantation

**DOI:** 10.1155/2011/432169

**Published:** 2011-08-29

**Authors:** Annelieke W. M. Paantjens, Ed A. van de Graaf, Johanna M. Kwakkel-van Erp, Tineke Hoefnagel, Walter G. J. van Ginkel, Farzia Fakhry, Diana A. van Kessel, Jules M. M. van den Bosch, Henny G. Otten

**Affiliations:** ^1^Department of Immunology, University Medical Centre Utrecht, 3584 EA Utrecht, The Netherlands; ^2^Department of Respiratory Medicine, University Medical Centre Utrecht, 3584 CX Utrecht, The Netherlands; ^3^Department of Pulmonary Disease, Antonius Hospital, 3430 EM Nieuwegein, The Netherlands

## Abstract

The production of IgG HLA antibodies after lung transplantation (LTx) is considered to be a major risk factor for the development of chronic rejection, represented by the bronchiolitis obliterans syndrome (BOS). It has recently been observed that elevated levels of IgM HLA antibodies also correlates with the development of chronic rejection in heart and kidney transplantation. This study investigates the relationship between IgM and IgG antibodies against HLA and MICA after lung transplantation. Serum was collected from 49 patients once prior to transplantation and monthly for up to 1 year after lung transplantation was analyzed by Luminex to detect IgM and IgG antibodies against HLA and MICA. The presence of either IgM or IgG HLA and/or MICA antibodies prior to or after transplantation was not related to survival, gender, primary disease, or the development of BOS. Additionally, the production of IgG alloantibodies was not preceded by an increase in levels of IgM, and IgM levels were not followed by an increase in IgG. Under current immune suppressive regimen, although the presence of IgM antibodies does not correlate with BOS after LTx, IgM^ high^ IgG^ low^ HLA class I antibody titers were observed more in patients with BOS compared to patients without BOS.

## 1. Introduction

The bronchiolitis obliterans syndrome (BOS) represents chronic rejection that accounts for the majority of mortality after lung transplantation (LTx). Almost 50% of lung transplant recipients develop BOS within five years after LTx [1–3]. BOS consists of damage and fibrosis within the airways, leading to decreased lung function which is used to diagnose chronic rejection after LTx [4]. Although the mechanisms underlying the pathogenesis of BOS remain unclear, several risk factors have been identified. The appearance of IgG antibodies against human leukocyte antigens (HLA) after lung transplantation is one of the major risk factors for chronic rejection [5–8].

The presence of IgG anti-HLA in patient sera reactive with antigens present on the donor lung prior to transplantation is a contraindication for transplantation [9, 10]. The majority of HLA diagnostics identify HLA antibody specificity prior to or after transplantation of the IgG isotype. The presence of this isotype is indicative of T-cell reactivity, as T cells are required to facilitate the isotype switch from IgM to IgG. Current immune suppressive regimens used after transplantation are focused on inhibiting T-cell function, including help for isotype switching [11]. We recently described the absence of IgG anti-HLA after lung transplantation when patients were treated with a tacrolimus/mycophenolate mofetil immunosuppressive regimen [12]. Therefore, we hypothesize that the low levels of IgG antibodies observed during this regimen may be the result of repression of IgM to IgG class switching. IgM antibodies develop early during the immune response and are able to fix complement efficiently. Although the presence of IgM antibodies prior to or after transplantation was initially considered to be harmless, it has recently been demonstrated that the presence of these antibodies in heart or kidney transplant patients may be predictive of rejection [13].

The goal of this study was to determine the relationship between IgM HLA antibodies after lung transplantation and the development of BOS. Additionally, we examined whether a correlation existed between IgM and IgG HLA antibodies after lung transplantation to determine if the isotype switch is inhibited by the immune suppressive regimen of tacrolimus/mycophenolate mofetil.

## 2. Methods

### 2.1. Patients

A total of 49 LTx patients transplanted between September 2003 and March 2008 at the Heart Lung Centre in Utrecht, The Netherlands, who exhibited a greater than three months survival, were included in this study. Eleven patients developed BOS during followup. BOS was defined as an irreversible decline in FEV_1_ of more than 20% compared to the postoperative baseline in the absence of infection or other etiology [14, 15]. Standard immunosuppressive therapy consisted of basiliximab, tacrolimus, mycophenolate-mofetil, and prednisone. No standard surveillance bronchoscopies were performed. In patients where a decline in lung function was observed, infections were diagnosed by cultures or BALF, and PCR was used for the diagnosis of CMV and EBV. When infections were excluded as the cause of FEV_1_ decline, patients were treated with corticosteroids and azithromycin.

The study design was approved by the medical ethical committee. Informed consent was obtained from each patient. Patients donated blood every month during the first year after transplantation and once every three months in the following years.

### 2.2. HLA Antibodies

Sera of 49 patients with known HLA type were screened for the presence of anti-HLA and anti-MICA antibodies prior to transplantation and then longitudinally, with an average of 20.7 months (range 10–29 months), after transplantation. A total of 382 samples were analyzed for IgG, and 477 samples were analyzed for IgM antibodies against HLA using Luminex beads according to the manufacturer's protocol (LABScreen Mixed, LSM12, One Lambda). For the IgM assay, the IgG detecting antibody was replaced with an IgM detecting antibody (R-phycoerythrin-conjugated AffiniPure F(ab) Fragment Donkey anti-human IgM obtained from Jackson ImmunoResearch). Three hundred eighty-two samples obtained from 49 patients were analyzed for both IgG and IgM.

The IgM tests were validated by measurement of 30 unimmunized males and measurements of samples obtained from kidney transplantation patients known to have high titers of IgM using both ELISA and Luminex. Optimal serum dilutions, conjugate dilutions, and incubation times were determined.

### 2.3. Depletion of IgM from Sera

Dithiothreitol (DTT) was used to deplete serum IgM as previously described [16]. Sera were incubated with an equal volume of 0.01 M DTT for 30 minutes at 37°C before testing as described above. A negative control (sample containing high levels of IgG and no IgM that was treated with DTT) and a positive control (sample containing high IgM and no DTT) were included in addition to sera that were diluted with PBS and not depleted of IgM by DTT.

### 2.4. Definition of Antibody Positivity

Positivity for IgG anti-HLA or -MICA was defined using the default settings of the software (Fusion) provided by the manufacturer (One Lambda). For IgM-positive HLA and MICA antibodies, a threshold was defined. Thirty healthy HLA-unimmunized males were analyzed to determine background levels. For each bead, the average background, including the range and standard deviation, was determined in these healthy controls. A patient was considered to have positive antibodies when the mean fluorescent intensity (MFI) (corrected for the negative bead) was >5 times the average background MFI of the unimmunized males.

Titers of IgM and IgG antibodies were defined as IgM^high^/IgM^low^ when MFI values were above or below the overall average of all beads in thirty healthy HLA-unimmunized males or IgG^high^/IgG^low^ when MFI levels were above or below the average background levels of all beads for IgG as defined by the manufacturer.

### 2.5. Possible Donor-Specific Antigens and Third-Party Antigens

For each patient, it was determined which beads contained at least 1 antigen from a mismatched donor HLA. These beads were considered to possess possible donor-specific antigens (DSA). Beads that were negative for mismatched donor HLA antigens were identified as third-party antigens (TPA).

### 2.6. Statistics

A *P* value of < 0.05 was considered to be significant. For correlation analysis, a *P* value of < 0.05 combined with −0.4 ≤*r*≥ 0.4 was considered significant. Correlations were analyzed using spearman rank correlation analysis, and differences in patient characteristics were analyzed by Mann-Whitney rank-sum, Fisher exact, or chi-square tests. These tests were also used to analyze differences in IgM and IgG titers between patients with and without BOS.

## 3. Results

### 3.1. Patient Characteristics

This study involved 49 patients whose details are displayed in Table 1. Four patients died as a result of BOS, and 1 patient died due to other causes. As expected, the mortality rate was higher in patients diagnosed with BOS compared to patients without BOS (*P* = 0.02, Fisher exact test). It should be noted that the group of BOS patients did not differ from the patients without BOS with respect to gender, age, primary disease, type of graft, ischemic time, or HLA mismatches for class I and class II. The average onset of BOS was 22.5 months after lung transplantation.

### 3.2. Presence of Antibodies before and after Transplantation

**Figure 1:**
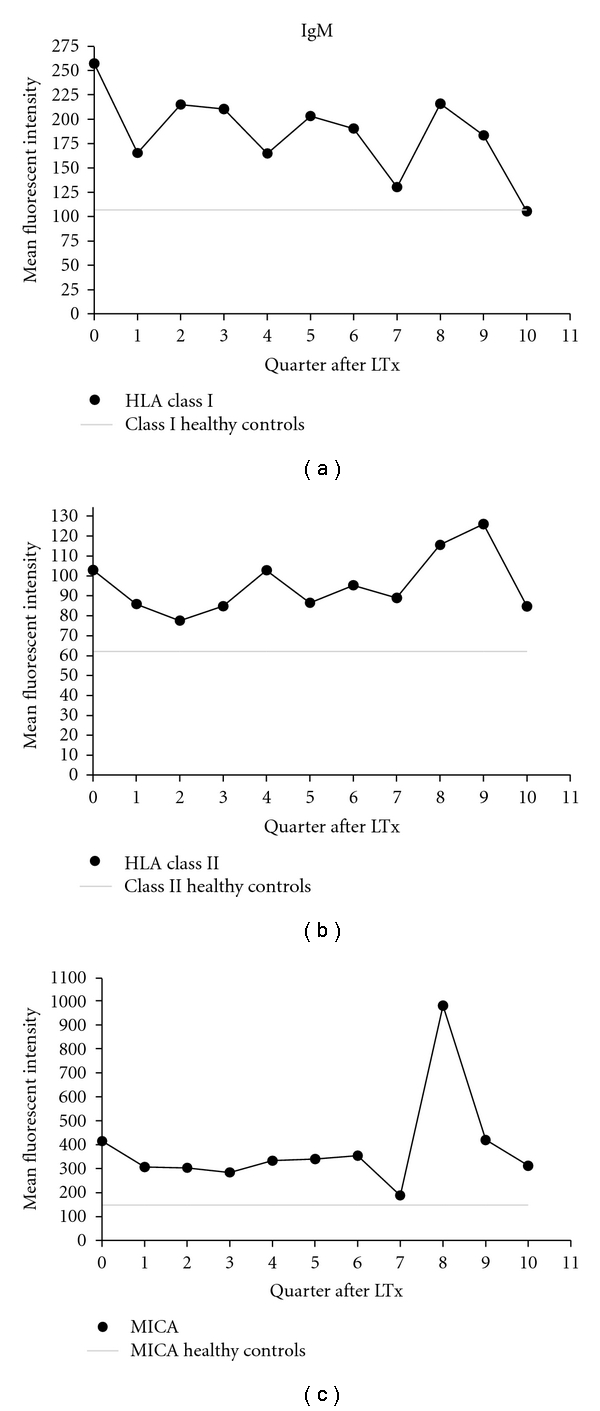
Average mean fluorescent intensity (MFI) of IgM isotype HLA class I, class II and MICA antibodies over time (a quarter is equal to 4 months). Forty-one patients were analyzed once prior to LTx, and 49 patients were measured longitudinally after transplantation resulting in a total of 477 samples that were analyzed by Luminex (LABScreen Mixed, LSM12, One Lambda, Calif, USA), with an average of 9.7 samples (range 5–10 samples) per patient. Shown are results of Luminex analysis with beads containing HLA class I (closed dot) class II (closed square), or MICA antigens (triangle with dotted line). These data were averaged from all LTx patients per quarter after lung transplantation.

We obtained serum from 41 patients prior to transplantation. Twelve of these patients tested positive for IgM anti-HLA class-I and/or class-II, and 5 patients tested positive for IgG HLA class I or II antibodies, indicating that IgM antibodies against HLA occurred more frequently in our study population compared to IgG antibodies. Additionally, antibodies against MICA were more frequently of the IgM isotype (*n* = 7) compared with the IgG (*n* = 2) isotype. After transplantation, serum was collected monthly from 49 patients. It was observed that antibodies against HLA class I and/or class II were more frequently of the IgG (*n* = 23) isotype than of the IgM (*n* = 16) isotype. MICA antibodies, however, were more frequently of the IgM (*n* = 14) isotype (IgG, *n* = 7). No relationship was observed between presence of antibodies before and after lung transplantation. The majority of patients who, prior to LTx, have antibodies against HLA or MICA also express these antibodies after transplantation. Some of these antibodies are not persistently reactive with the same HLA-coated Luminex beads, and some patients expressing antibodies prior to LTx do not express them after LTx. In 25 patients, de novo antibodies against HLA or MICA appeared after transplantation, with an average first appearance after transplantation at 7.5 months (HLA class I), 17.6 months (HLA class II), and 8.4 months (MICA). A longitudinal analysis was performed to determine if specific patterns in the elevation or reduction of titers could be observed. Although, on average, low but stable titers of HLA class I, class II, and MICA IgM antibodies were detected after LTx, average titers were elevated compared to background titers (Figure 1). The MFI levels of 30 healthy unimmunized males were used to establish the background levels. For HLA class I, an average background MFI of 107 was observed, while LTx patients on average exhibited an MFI of 186. For HLA class II, a background level MFI of 62 was observed, while the LTx patient average was an MFI of 96. MICA titers were highest in LTx patients as compared to HLA classes I and II. MFI was 385 in LTx patients compared with a background of MFI 148 observed in unimmunized healthy males. MFI levels of the 3 antibody groups were relatively stable after LTx. Although a peak in the levels of MICA antibody was observed in the 8th quarter after LTx, this was the result of a low sample number in that quarter and 1 patient exhibiting a high titer of MICA antibodies after LTx. 

### 3.3. Correlation between IgM and IgG Antibodies and Isotype Switching

**Figure 2:**
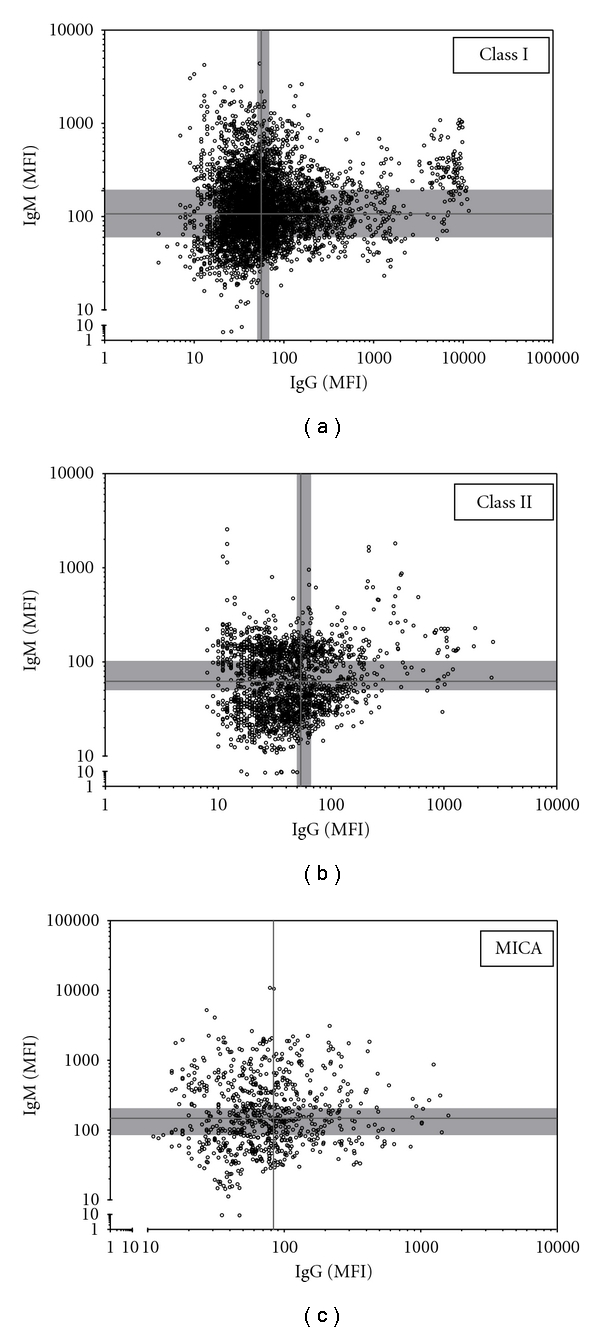
Correlation plots of IgM and IgG HLA class I (a), HLA class II (b), and MICA (c) antibodies after LTx. Grey lines represent the average background as detected in 27 unimmunized males (IgM) or as defined by the manufacturer (IgG). The background range of the individual beads is displayed by the grey area.

A total of 341 serum samples taken after LTx were analyzed for both HLA and MICA antibodies of IgM and IgG isotype, with a total of 12,958 bead-serum combinations being measured. The MFI values of beads containing either HLA class I, HLA class II, or MICA antigens are displayed in Figure 2, with each dot representing MFI values derived from IgM/IgG assays of 1 Luminex bead tested against 1 serum. Average background values for IgM and IgG are displayed as grey bars, with each graph divided into four quadrants. In Figure 2(a), the correlation between IgM and IgG for HLA class I after transplantation is indicated by 8,182 bead measurements. Most samples titers of IgM and IgG are near background levels. Although some sera show elevated titers for either IgM or IgG; almost no sample is positive for both IgM and IgG. A small percentage of the data points did exhibit elevated IgM and extremely elevated IgG MFI values; however, these data were all derived from the same individual. This was a female (40 years) who already exhibited elevated MFI values prior to transplantation and had also experienced two pregnancies. As fewer beads in the kit are coated with HLA class II or MICA antigens, graphs depicting the results of these studies contain a reduced number of data points (Figures 2(b) and 2(c), resp.). In agreement with previous results, samples containing both IgM and IgG antibodies against HLA class-II antigens were rare. This was also observed in studies of antibodies against MICA. These findings indicate no correlation between IgM and IgG levels of anti-HLA class I, II or MICA antibodies after LTx. The majority (approximately 80%) of samples exhibit an average or low MFI compared to the average background observed in unimmunized controls (Figures 2(a), 2(b), and 2(c)).

To investigate whether the lack of positivity for IgG anti-HLA antibodies may result from the physical inhibition by IgM anti-HLA antibody binding to the beads, 10 sera samples exhibiting elevated levels of MFI specific for IgM anti-HLA class I antibodies were selected and treated with DTT to disrupt IgM. One sample exhibiting high IgG MFI levels and low expression of IgM was used. IgM anti-HLA was not detected following incubation with DTT, and MFI levels of IgG anti-HLA did not increase. The IgG anti-HLA control sample still exhibited elevated MFI values following DTT treatment, indicating that IgG binding was not impaired by DTT (data not shown). Given these findings, the lack of IgG anti-HLA detection in serum samples with IgM anti-HLA antibodies is not the result of inhibition by antibodies of the IgM isotype.

**Figure 3:**
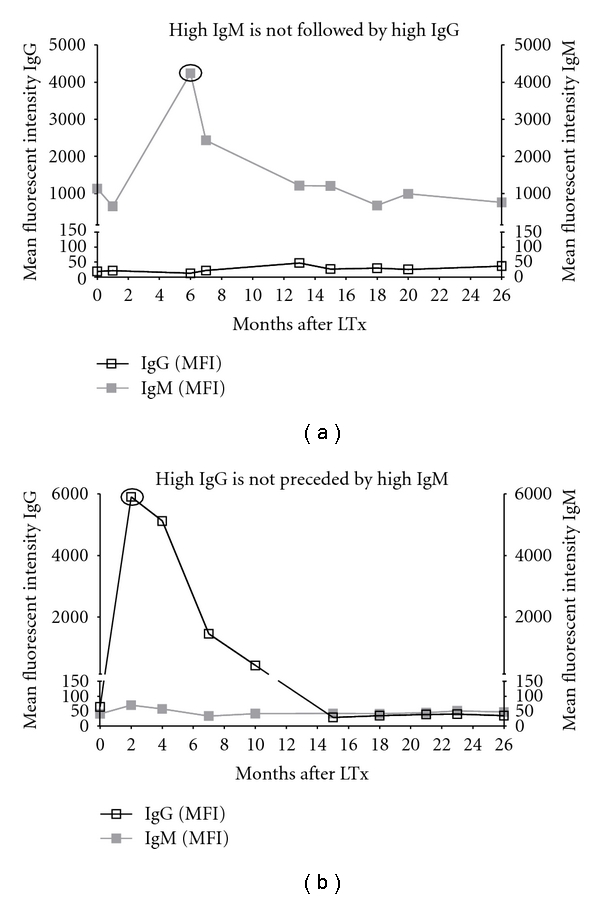
Examples of no correlation between levels of IgM and IgG anti-HLA. Elevated levels of IgM are not followed in time by high levels of IgG levels (a), and increased levels of IgG are not preceded by high levels of IgM (b). Figures are indicative of 1 bead obtained from 1 patient in time.

In samples containing elevated levels of IgM or IgG antibodies against HLA, it was investigated whether high IgM was followed in time by high IgG or whether high IgG was preceded by high IgM. To this end, beads were identified recognized by patient sera and the other sera collected from the same patients at other time points were analyzed for reactivity against the same beads. Figure 3 illustrates a longitudinal analysis recognition of a specific bead by IgM and IgG anti-HLA antibodies.  Figure 3(a) provides an example of high IgM levels which are not followed by IgG through the means of isotype switching, as IgG levels remain very low.  Figure 3(b) indicates that elevated levels of IgG are not preceded by high IgM expression. Longitudinal analysis of all sera exhibiting either high IgM or IgG MFI values provided comparable results, indicating that, in these samples of lung transplant patients; there is no relation between IgM and IgG antibodies against HLA or MICA.

### 3.4. Relation between Clinical Parameters, Antibodies, and Antibody Titers

We next investigated if clinical characteristics were related to IgM or IgG anti-HLA antibodies present prior to lung transplantation. There was no difference in the gender of patients that tested positive for IgM or IgG isotype HLA antibodies prior to transplantation. The percentage of males expressing IgM antibodies against MICA prior to transplantation was slightly higher than that of females (*P* = 0.09, fisher exact test). Patients lacking IgM antibodies were younger than patients expressing MICA IgM antibodies (*P* = 0.04, Mann-Whitney rank-sum test). Transplant patients suffering from end-stage cystic fibrosis prior to LTx were younger than patients diagnosed with emphysema or fibrotic diseases. These cystic fibrosis patients exhibited reduced levels of IgM antibodies compared to the other patients (*P* = 0.014, chi-square) and, also, showed a reduction in HLA class I and/or class II IgM antibodies (*P* = 0.002, chi square). These differences in clinical characteristics in patients with and without IgM antibodies were not observed after lung transplantation.

**Figure 4:**
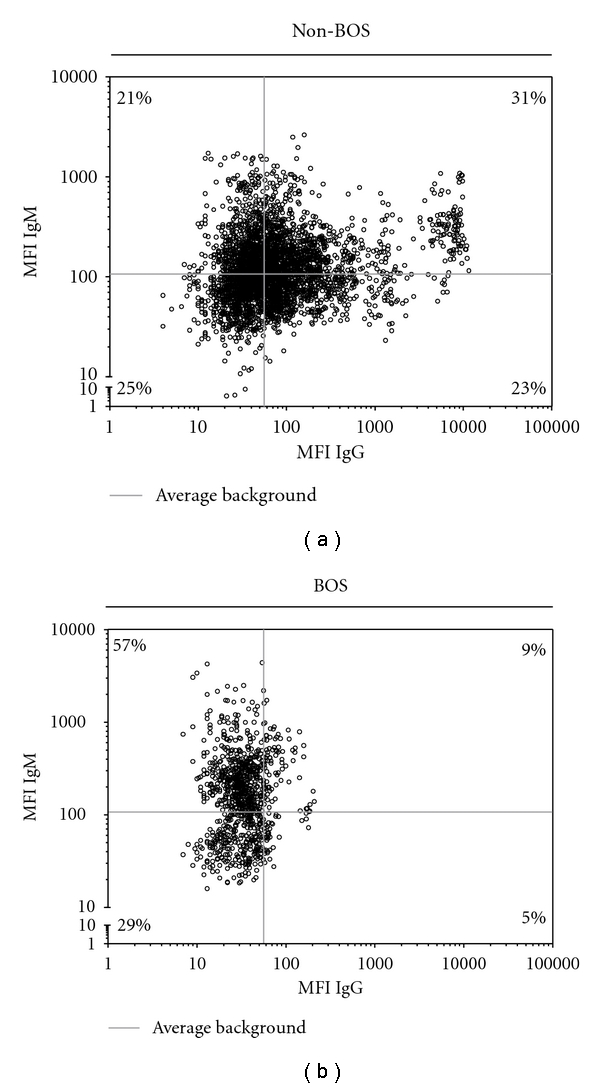
Differences in HLA class I antibody isotype profiles in BOS and non-BOS patients.

**Figure 5:**
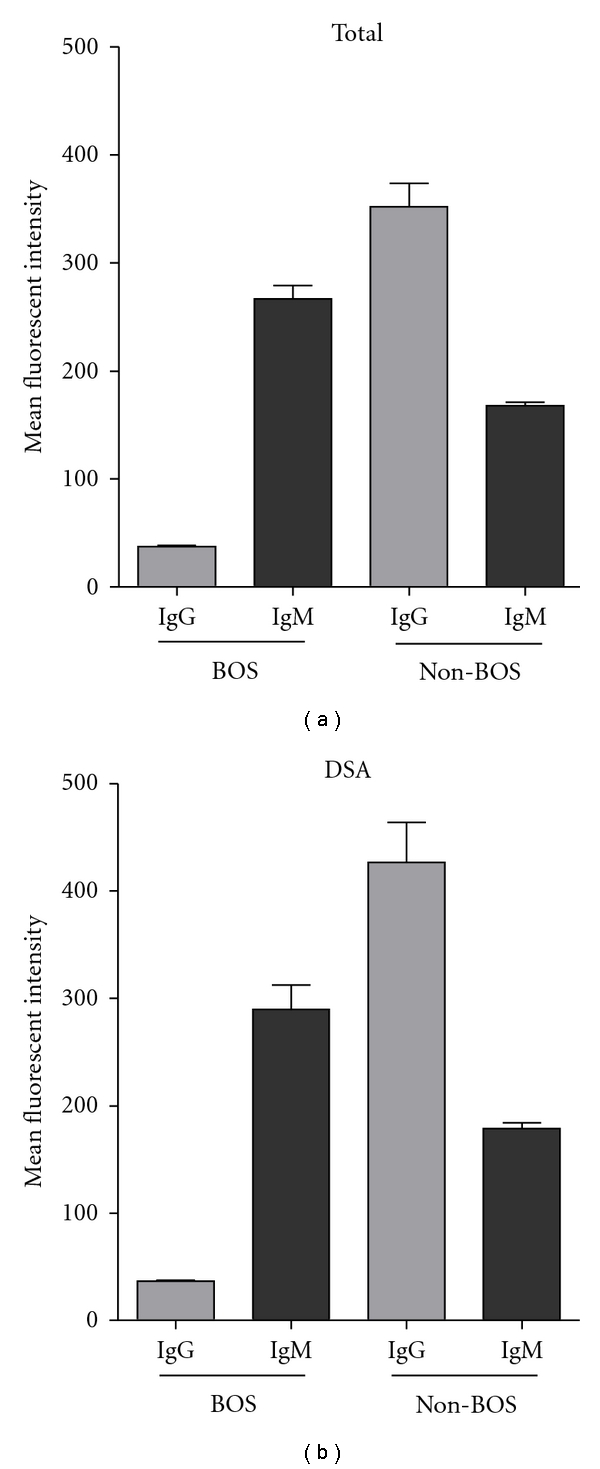
Mean fluorescent intensities of HLA class I antibodies. (a) All HLA class I antibodies of both IgG and IgM isotype in BOS and non-BOS patients. (b) Possible DSA HLA class I antibodies of both IgG and IgM isotype in BOS and non-BOS patients. Error bar depicts the standard error of mean.

Analysis of antibody titers after LTx indicated no relationship to the development of BOS (data not shown), as no rise or fall in antibody titers was observed prior to BOS diagnosis. The presence of IgM or IgG antibodies against HLA class I, HLA class II, and/or MICA prior to or after lung transplantation is not related to the development of BOS. Donor-specific antibodies may better correlate with development of BOS, so we designated DSA for beads with possible donor-specific antigens and TPA beads lacking these antigens. Our results indicated no differences between patients with BOS and without BOS for IgM anti-HLA, although a trend was observed in patients lacking IgG anti-HLA, as they developed BOS with a higher frequency (*P* = 0.06, Kaplan Meyer test; data not shown). To examine whether differences observed between BOS and non-BOS patients were correlated with antibody titers, all data points were plotted in separate correlation graphs. We observed that the majority of the samples obtained from patients eventually diagnosed with BOS were below IgG background levels (Figure 4(b)), while elevated antibody titers were detected in patients that do not develop BOS (Figure 4(a)). By contrast, patients diagnosed with BOS had relatively more samples with high IgM titers (Figure 4(b)) compared to non-BOS patients (Figure 4(a)). To quantify this, we determined the number of samples possessing IgM^low^ IgG^low^, IgM^high^ IgG^low^, IgM^low^ IgG^high^, or IgM^high^ IgG^high^ antibody titers. In BOS patients, 66% of the samples were IgM^high^ while 52% samples of patients without BOS had high IgM titers (*P* = 0.04, fisher exact test). Differences in IgG antibody titers were more defined, with only 14% of BOS patients exhibiting elevated IgG antibody titers versus 54% of the samples from non-BOS patients (*P* ≤ 0.0001, fisher exact test). For HLA class II, there was no observable difference between BOS and non-BOS patients. Analysis using DSA and TPA coated beads also did not indicate any differences. In Figures 5(a) and 2(b), the mean fluorescent intensity of IgG or IgM isotype HLA class I antibodies is displayed, showing that there are no differences between total of HLA class I antibodies and the possible DSA HLA class I antibodies. However, differences between BOS patients and non-BOS patients become clear; an increase is seen in the titers of IgG HLA class I antibodies of non-BOS patients compared to BOS patients, while a decrease is shown for titers of IgM HLA class I antibodies of non-BOS patients compared to BOS patients. 

## 4. Discussion

In this study, we demonstrate that the presence of IgM HLA and MICA antibodies prior to or after lung transplantation is not related to the development of BOS. We also show that the course of IgM antibody titers is stable after lung transplantation. After LTx, there is no correlation between IgM and IgG antibodies, as high IgM is not followed by high IgG and high IgG is not preceded by IgM. Patients diagnosed with BOS, however, do exhibit elevated HLA class I IgM antibody titers and low IgG titers compared to patients without BOS.

IgM antibodies against HLA have been considered to be clinically irrelevant, but recent findings challenge this concept. In heart transplantation, it was shown that non-HLA antibodies of IgM isotype were correlated with a reduction in graft survival [13, 17]. In the context of heart and kidney transplantation, it has also been demonstrated that expression of HLA antibodies of the IgM isotype correlates with transplant rejection [13]. The sensitive Luminex method used in this study made it possible to detect the presence of low HLA IgM antibody titers. Consistent with the results of HLA IgG antibody studies, no relationship could be detected between the presence of low HLA IgM antibody titers and chronic rejection or overall survival in patients treated with an immune suppressive regimen consisting of tacrolimus and mycophenolate mofetil after lung transplantation [12]. In concert with this, a study of kidney transplantation also showed that low titers of DSA HLA IgG antibodies present prior to transplantation are not detrimental to overall survival and do not correlate with acute or chronic rejection [18]. 

One study detected a correlation between MICA antibodies and development of rejection after kidney transplantation. However, a number of other studies of heart or kidney transplantation did not find such a relation [19–22]. Although MICA antibodies appear to contain minor complement fixing abilities, MICA is expressed in high levels in the renal system and intestines and is not detected in the heart or aorta, raising the possibility that the ability of these antibodies to bind complement to induce antibody-mediated rejection is organ specific [22]. Although MICA expression can be induced on airway epithelial cells by stress, we observed no relationship between the presence of MICA antibodies and the chronic rejection of graft survival. In our study, MICA antibodies were present in both males and females prior to transplantation, although levels were slightly higher in males. These results and previous studies indicate that it is unlikely that MICA antibodies develop due to pregnancy.

High levels of IgM antibodies in serum may physically inhibit the binding of IgG antibodies to the Luminex beads [23]; however, experiments using DTT treatment indicated that IgM antibodies could be depleted from the serum and that MFI levels of IgG did not increase. This indicates that the lack of IgG^high^ antibody titers in BOS patients is not an artifact caused by high concentrations of IgM antibodies. Elevated levels of IgG anti-HLA that are detected after lung transplantation are also commonly observed prior to transplantation. The production of these antibodies is therefore not hampered by the immunosuppressive treatment with tacrolimus and mycophenolate mofetil administered after transplantation. We also observed no indication of IgM replacement by IgG alloantibodies, suggesting a lack of class-switching of B cells that may be due to the immunosuppression of T cells. These findings revealed that de novo IgG anti-HLA antibodies after lung transplantation yielded low levels of MFI.

Although titers of HLA class I IgM^high^ IgG^low^ antibodies are elevated in patients with BOS, this cannot be used as a possible marker for the development of BOS because some patients without BOS also have antibody titers exhibiting elevated IgM and low IgG. Additionally, although patients without BOS express higher HLA IgG antibody levels compared to patients with BOS, some considerations have to be placed. First, HLA IgG antibody titers are low and may not be capable of activating the complement cascade. Second, it is unknown if IgG HLA antibodies are of the IgG1, IgG3 subclasses able to fix complement, or of the IgG2 and IgG4 subclasses. If HLA antibodies are sequestered to the transplanted lung rendering them undetectable in BOS patients, they could fix the complement system and hence contribute to the development of BOS after lung transplantation [13, 24, 25]. Furthermore, there are reports that low titers of HLA antibodies may be protective instead of harmful [22, 26]. It is also unclear if the relationship between BOS and HLA class-I antibody levels contributes to the disease process or is an epiphenomenon. In our assays, we used beads with multiple antigens coated on them which did not allow us to detect specific donor reactivity. It should be noted that using beads specific to either DSA or TPA did not reveal any significant difference, and therefore a test with single antigen beads probably does not contribute to our findings. We also could not exclude cross-reactivity between the antibodies.

In conclusion, lung transplant patients develop IgM HLA and MICA antibodies prior to and after transplantation. The presence of these antibodies and their titers are not related to chronic rejection under the current immune suppressive regime, and the isotype switch is inhibited. Patients diagnosed with BOS, however, exhibit elevated levels of IgM and reduced IgG HLA expression compared to patients without BOS. HLA antibodies are thought to be involved in chronic graft rejection as a result of their complement fixing ability as observed by C4D deposition within the graft. Mechanistically, our data could imply that IgM HLA antibodies are relevant to the pathogenesis of BOS because they are able to fix complement more efficiently than IgG.

## Figures and Tables

**Table 1:** Patient characteristics.

	BOS	Non-BOS
Total number	11	38
Deceased	4 (36%)	1 (3%)
Gender		
Male	3 (27%)	22 (58%)
Female	8 (73%)	16 (42%)
Average age (years; SD)	45 (+15)	42 (+13)
Primary disease		
CF	3 (27%)	19 (50%)
EMF	5 (46%)	9 (24%)
FD	3 (27%)	10 (26%)
Type of graft		
Single	2 (18%)	4 (11%)
Bilateral	9 (82%)	34 (89%)
Average ischemic time (minutes; SD)		
Single	216 (±70)	209 (±43)
Bilateral	317 (±51)	322 (±72)
HLA mismatches (average; SD)		
Class I	3.6 (±0.7)	3.1 (±0.7)
Class II	1.7 (±0.5)	1.7 (±0.5)
BOS onset (months; SD)	22.5 (±13.9)	ND
BOS grade		
I	3 (27%)	ND
II	2 (18%)	
III	6 (55%)	

BOS: bronchiolitis obliterans syndrome, SD: standard deviation, CF: cystic fibrosis, EMF: emphysema, and FD: fibrotic disease.
